# Somatics of Early Buddhist Mindfulness and How to Face Anxiety

**DOI:** 10.1007/s12671-020-01382-x

**Published:** 2020-05-08

**Authors:** Bhikkhu Anālayo

**Affiliations:** Barre Center for Buddhist Studies, 149 Lockwood Road, Barre, MA 01005 USA

**Keywords:** Anxiety, COVID-19, Fear, *kāyagatāsati*, Mindfulness of the body, Proprioception, Somatics

## Abstract

The body is a central object of the cultivation of mindfulness, in the way this has been described in relevant Pāli discourses and their parallels. At the background of such cultivation stands the absence of positing a mind-body duality and a lack of concern with a particular physical location of the mind in early Buddhist thought. Moreover, the various exercises that involve directing mindfulness to the body need to be considered in conjunction in order to arrive at a balanced understanding of their overarching purpose. Out of the different possible modalities of cultivating mindfulness in this way, the discourses present awareness directed to one’s own bodily postures as a practice already undertaken by the Buddha-to-be when he was still in quest of awakening. In this particular setting, such mindfulness of postures served as a way of facing fear. The potential of this exercise to provide a grounding in embodied mindfulness, being fully in the here and now, is of particular relevance to the challenges posed by the current pandemic.

At the background to the role of the body as an object of mindful contemplation stands the way the body-mind relationship is conceived in early Buddhism. Another important consideration concerns the need to evaluate individual exercises for contemplating the body within their context, for which purpose a survey of practices provided in the Discourse on Mindfulness of the Body can offer a convenient starting point.

Beginning with the first of these topics, early Buddhist thought does not subscribe to a body-mind dualism of the Cartesian type, but much rather envisages the continuity of subjective experience as involving a reciprocal conditioning between consciousness on the one side and mental activities (subsumed under the header “name,” in the sense of those functions that lead to naming things) together with the experience of materiality (referred to under the header “form”) on the other side (Anālayo 2019b). In other words, rather than dividing experience into what is material and what is mental, this mode of presentation draws the line between the process of receptively knowing, of being conscious, and the conceptual and material aspects of experience that furnish the content of what consciousness is aware of.

## Relating the Mind to the Body

Given this basic premise, it is perhaps not surprising that early Buddhist thought does not exhibit a conspicuous concern with the precise physical location of the mind. The brain receives very little attention, to the extent that it is not even included in a listing of bodily parts in the *Satipaṭṭhāna-sutta*’s instructions for mindful contemplation of the body’s anatomy (MN 10). Only some parallel versions and texts reflecting later tradition add the “brain” (*matthaluṅga, mastakaluṅga,* 腦, *klad pa*) to such listings (Anālayo 2018c, p. 151).

Nevertheless, the brain was clearly known in ancient India (e.g., Sn 199). The Pāli *Vinaya* even describes what by all means appears to be a case of successful brain surgery by the physician Jīvaka, who regularly attended to the Buddha and his monastic disciples (Vin I 274; Zysk 1982). Thus, the omission of the brain in listings of anatomical parts is not due to a lack of knowledge of the existence of this part of the body. Instead, it rather points to an absence of interest in the brain, probably reflecting insufficient awareness of its role in relation to the mind.

In modern parlance, the brain tends to be regularly mentioned when intending to refer to the mind. Examples are usage such as “this is a no-brainer,” “pick someone’s brain,” “a brainstorm,” “have something on the brain,” “to wrack one’s brain,” and to be “bored out of one’s brains.” A comparative usage in early Buddhist texts rather involves the “heart.” An example in case is a description in the *Anaṅgana-sutta* of a former cartwright watching another cartwright planing a felloe. When the latter does this exactly as the former would have wished, the former exclaims that the planing was done in such a way that “one would think [he did it] as if knowing [my] heart with [his] heart” (MN 5: *hadayā hadayaṃ maññe aññāya*).

In Chinese parallels to this discourse, this reflection is about the other cartwright “knowing my mind” (MĀ 87: 知我心), “knowing my intention” (T 49: 知我意) or acting “according to my intention” (EĀ 25.6: 如我意). The Chinese term 心, used in the *Madhyama-āgama* parallel listed first, translates both “mind” and “heart.” It occurs in the *Madhyama-āgama* parallel to the *Satipaṭṭhāna-sutta* in the listing of anatomical parts to refer to the heart as an organ and again in the same discourse’s description of the third establishment of mindfulness to refer to different mental states (MĀ 98). Hence, the reference to “knowing my mind” in the present case could well be based on an original term equivalent to Pāli *hadaya.*

The same holds for the next example, which concerns a recurrent reference to *hadaya* in Pāli discourses when describing agreeable ways of speaking. One of the ways in which speech can be pleasant, in contrast to being harsh, is when it quite literally “goes to the heart” (MN 27: *hadayaṅgama*), which the Chinese parallel expresses as “entering the mind” (MĀ 146: 入心).

Another example occurs as part of a Pāli stanza, which describes having “removed afflictions from the heart” (SN 10.8: *vineyya hadaye daraṃ*), in which case the Chinese parallels speak of having “subdued the fevers of the mind” (SĀ 592: 調伏心熾燃) or of having “removed feverish afflictions from the mind” (SĀ^2^ 186: 心除熱惱病).

These few examples suffice to show that, as far as relating the mind to some particular part of the body is concerned, early Buddhist texts tend to choose the heart rather than the brain. Later Theravāda tradition then goes further by positing the “heart-base” (*hadayavatthu*) as the physical basis for the mind (Vism 447; Karunadasa 2010, p. 79).

Of further interest for the overall topic of the body-mind relationship is also a usage of the Pāli term for body (*kāya*) in a way that stands representative of the whole of one’s “personal experience” (Anālayo 2011, p. 379). Grammatically speaking, this involves the instrumental *kāyena*, literally “by way of the body.” This expression can be used to describe the experience of the immaterial attainments, even though these are devoid of materiality and thus cannot involve the physical body. Nevertheless, some early discourses depict their attainment as involving what literally would be a “touching by way of the body” (MN 70: *kāyena phusitvā/phassitvā* and MĀ 195: 身觸; the latter refers to the whole set of eight liberations, which include the immaterial attainments). The discourse parallels showing this type of employment also testify to another such usage, namely in relation to attaining the final goal, in the sense of realizing the truth “by way of the body” (MN 70: *kāyena … sacchikaroti* and MĀ 195: 身作證).

Such instances subsume the whole of mental experience under the same term “body” (*kāya*) that in the *Satipaṭṭhāna-sutta* and its parallels serves to designate the first establishment of mindfulness, which is concerned with different meditative perspectives on the physical body (MN 10, MĀ 98, and EĀ 12.1). This usage, conveying direct and personal experience, ties in well with what already emerged from the passages surveyed earlier regarding the way early Buddhist thought approaches the issue of the body-mind relationship.

## Ancient Indian Asceticism

A distinct Buddhist perspective on the role of the mind/heart, if this expression can be used, in relation to the body becomes further apparent when taking into consideration the ancient Indian context. Early Buddhist thought stands out in its historical setting for an emphasis on the mind and intentionality. This can be seen, for example, in a debate with a follower of the Jain tradition, reported in the *Upāli-sutta* (MN 56 and MĀ 133). Regarding the relative importance of bodily, verbal, and mental activities in relation to unwholesome and reprehensible deeds, according to this discourse, the representative of the Jain tradition took the position that bodily activities are the most important of these three. This might at first sight seem obvious, simply because bodily activities can inflict greater harm and hence need above all to be restrained. Yet, the Buddha disagreed, instead according chief importance to mental activities. The position taken in this way reflects the overarching concern with intentionality in early Buddhist ethics. Viewed from this perspective, bodily infliction of harm is ultimately due to harmful mental intentions that lead to reprehensible physical acts.

Of interest in this respect is also another discussion between the Buddha and a Jain debater, reported in the *Mahāsaccaka-sutta* (MN 36 and Liu 2010). The exchange begins with the debater distinguishing between cultivation of the body and cultivation of the mind (*kāyabhāvanā* and *cittabhāvanā*), explaining the first of these to stand for a range of different ascetic practices. When questioned by the Buddha about cultivation of the mind, however, it turned out that the debater had no clear idea of what this could imply. This neatly reflects the importance attached in the ancient Indian setting to the practice of asceticism for progress to liberation, combined with a lack of awareness of an alternative approach that rather places the focus on cultivating the mind.

The same pattern is similarly evident in the encounter between the recently awakened Buddha and those who were to become his first five disciples, reported in the *Ariyapariyesanā-sutta* (MN 26 and MĀ 204). These five had been the companions of the Buddha-to-be during his own practice of asceticism. Once he realized that ascetic practice was not conducive to liberation and abandoned it, these five left him in the belief that he had forsaken the path to awakening. According to the *Ariyapariyesanā-sutta* and its parallel, the Buddha did not find it easy to persuade these five that abandoning ascetic practice could actually be part of a path to awakening, rather than automatically implying a reversion to a life of sensual indulgence. The episode shows how deeply the belief that liberation requires physical mortification was ingrained in the ancient setting.

The overarching importance accorded to asceticism among ancient Indian practitioners must also have been the reason why the Buddha-to-be himself had undertaken practices like breath control and fasting in the first place (Anālayo 2017c). Although he eventually found these to be unconducive to liberation, he did incorporate aspects of each of these two practices in his later teachings, with the decisive difference of according chief importance to the mind in a way that gives a central role to mindfulness.

For example, monastic disciples of the Buddha and lay people on special observance days undertake intermittent fasting, in the sense of not taking food after noon. In addition, a basic practice relevant to both is the cultivation of mindfulness when eating, combined with the clear awareness that the purpose of taking food is to nourish the body rather than to entice the taste buds. From the viewpoint of contemporary mindfulness employment in healthcare and related areas, of particular interest is an instruction given by the Buddha to an overweight king on the cultivation of mindfulness for knowing moderation with food, an instruction that resulted in the king successfully reducing his weight (Anālayo 2018a, b).

In the case of the breath, a comparable shift in perspective can be discerned. Rather than attempting to control the breath, as the Buddha-to-be had attempted prior to his awakening, the early discourses offer detailed instructions on how to cultivate mindfulness of the natural process of breathing (Anālayo 2019c). The overall concern of these instructions is not to change the breath, but rather to be mindful of the way it occurs of its own accord.

## The Four Establishments of Mindfulness

The early Buddhist perspective on the body-mind relationship is also evident in instructions on the four establishments of mindfulness. Of particular interest here is that detailed instructions on mindfulness of breathing in altogether sixteen steps of meditation practice are explicitly shown to cover all of the four establishments of mindfulness (Anālayo 2019d). In this way, the breath, which in itself is a bodily phenomenon, as a meditative object relates not only to the first establishment of contemplation of the body but can also be used to cover the areas of feeling tones, mental states, and dharmas. Moreover, the same practice of being mindful of the breath can in turn lead on to a cultivation of the awakening factors, a central theme in the fourth establishment of mindfulness, contemplation of dharmas (Anālayo 2013).

Besides being the meditative culmination point of a form of practice based on the bodily phenomenon of the breath, a cultivation of the awakening factors can in turn have an effect on the body. This emerges in accounts of how the recitation of these awakening factors—which can safely be assumed to have prompted a corresponding meditative practice by the listener—brought about recovery from physical disease (Anālayo 2015, 2017b). Such potential is based in particular on mindfulness, which as the first among the seven awakening factors has a foundational role for the cultivation of the other six. In this way, the early discourses already recognize a specific physical healing potential accessible through the cultivation of mindfulness.

## The First Establishment: Mindfulness of the Body

Of the terrain covered by the four establishments of mindfulness, the body stands out for being mentioned repeatedly on its own elsewhere as an object for mindfulness practice. In fact, the same discourse collection that houses the *Satipaṭṭhāna-sutta* also has an entire discourse dedicated to this topic, which is the Discourse on Mindfulness of the Body (MN 119 and MĀ 81). The exercises common to the two parallel versions of this discourse cover the following (adopting the sequence in MN 119):

- Mindfulness of breathing,
- Mindfulness of the four postures,
- Mindfulness and clear knowing during various bodily activities,
- Mindfulness of the anatomical constitution of the body,
- Mindfulness of the physical constitution of the body (in terms of its elements),
- Mindfulness of the decay of the body after death,
- Mindfulness of the bodily dimension of the experience of the four absorptions.

Considering these different practices in conjunction relates to the earlier-mentioned need to evaluate individual exercises for contemplating the body within their context. This can be helpful to counter the impression of some scholars that the Buddhist attitude toward the body is a predominantly negative one. For example, according to Kajiyama (1972, p. 257), in the Buddhist tradition “the body is regarded as the origin of disgust, pain and evil, and emancipation from the body is the ideal.” Or else Faure (1998, p. 32) proposed that “Buddhist deprecations of the body, a fortiori the female body, aim at provoking a holy horror of sensual desire.”

One of the exercises listed above, contemplation of the anatomical constitution of the body, does indeed come with a distinctly negative evaluative element. However, this needs to be considered in relation to the other exercises described in the same discourse (Anālayo 2017a). Viewed within this context, it becomes clear that the purpose of this practice is not to provoke some sort of holy horror or else to come to regard the body as the origin of all evil. Instead, contemplation of the body’s anatomy can help to overcome obsession with sensuality, which in turn enables attaining the absorptions, whose bodily experience of intense rapture and happiness the very Discourse on Mindfulness of the Body depicts in detail and illustrates with the help of a set of similes.

## Mindfulness of Postures and Bodily Activities

Somewhere midway between the two poles that emerge in this way, where on the one hand mindfulness of the body serves to challenge perceptions of sexual attraction and on the other hand relates to profound levels of rapture and happiness on the bodily level, stand the exercises concerned with the postures of the body and its activities. These neither involve a negative evaluation of the body, nor do they, on their own, result in the intense rapture and happiness of absorption. In this intermediate position, they offer a direct and easily accessible way to inhabit the body, to cultivate an embodied type of mindfulness. This can be considered a way of expressing, on a very practical level, a central dimension of the body-mind relationship in early Buddhist thought.

The importance of an embodied mindful presence can be explored by turning to two similes whose explicit intention is to illustrate the topic of mindfulness of the body. One of these two illustrations describes a person who has to carry a bowl full to the brim with oil through a great crowd watching a dancing and singing performance. The carrier is followed by another person with a drawn sword, ready to cut off the carrier’s head if even a drop of the oil should be spilled. The depiction of this dramatic scene leads to the Buddha posing the following question to his audience:

What do you think, monastics, will that person, out of negligence, direct attention to the outside and not to that bowl of oil?

(SN 47.20: *taṃ kiṃ maññatha, bhikkhave, api nu so puriso amuṃ telapattaṃ amanasikaritvā bahiddhā pamādaṃ āhareyyā ti?*).

Monastics, what do you think, will that person carrying the bowl of oil be able to be unmindful of the bowl of oil, unmindful of the executioner, and look at that dancing girl and at the great crowd?

(SĀ 623: 云何, 比丘, 彼持油鉢士夫能不念油鉢, 不念殺人者, 觀彼伎女及大眾不?).

Of course, the carrier will not do that and will instead pay attention continuously to the bowl to prevent spilling any oil and thereby avoid being executed. At the same time, however, such paying of attention would not involve a strong and exclusive focus on just the bowl. The carrier needs to have at least peripheral awareness of the crowd’s movements to be able to walk through without bumping into someone and thereby losing balance. In other words, there is a need to stay attuned to the environment in order to be able to maintain the bodily balance required for keeping the bowl similarly balanced.

A central implication of this simile appears to be the potential of whole-body awareness, such as can be cultivated with mindfulness of postures and of bodily activities. This type of mindfulness deployment can provide a tool to establish a firm grounding in the presence of the body. Such practice strengthens continuity of mental presence and allows combining the task of close attention to the bowl with an overall monitoring of the entire situation.

A similar nuance emerges in another simile, where mindfulness of the body finds illustration in a strong post to which different animals, representing the senses, are bound (SN 35.206, SĀ 1171, EĀ 38.8, and Up 9006). However much these animals struggle to escape in one direction or another, due to being firmly bound to the post they are unable to do so. This illustrates the potential of mindfulness of the body to provide an element of centering that counters the tendency toward a fragmentation of experience, which easily happens when the mind follows whatever happens to be the most prominent object at any of the senses. With mindfulness of the body established, it becomes possible to remain centered and balanced, regardless of what happens at any sense door. This complements the imagery of carrying a bowl of oil, showing that a key aspect of mindfulness of the body can be found in its potential to establish a centering element in challenging circumstances.

Out of the exercises surveyed in the Discourse on Mindfulness of the Body, the practices directly relevant to these two similes appear to be mindfulness of postures and of bodily activities. The type of proprioceptive awareness that can be cultivated with the help of these practices would be most pertinent to the task of the carrier of the oil, illustrative of the need to remain centered amidst the tendency of the senses to pull and push the mind in one direction or another.

The Discourse on Mindfulness of the Body continues from its survey of different modalities of mindfulness of the body to providing a long list of the potential benefits of such practice. The benefits listed range from enduring or overcoming fear and discontent, as well as being able to bear with bodily afflictions caused by weather and hunger or thirst, to the attainment of the absorptions and the stages of awakening. Out of these benefits, the topic of fear is of particular relevance to the present worldwide situation and thereby offers a convenient entry door into applying the early Buddhist perspective on the body-mind relationship and related mindfulness practice to the current pandemic and its challenges, in particular the need of learning how to face fear and anxiety.

## Mindfulness and Fear

The four postures of walking, standing, sitting, and lying down occur in an account of the practices undertaken by the Buddha-to-be when he was still in quest of awakening. The whole discourse, in which this episode occurs, is about the topic of fear. This thereby helps to relate the benefit of enduring or overcoming fear, mentioned in the Discourse on Mindfulness of the Body, to one of the practices described in the same discourse, namely, mindfulness of postures. Turning from that discourse to the report of the practices undertaken by the Buddha-to-be, the relevant passage describes how he dwelt in forest solitudes and at times heard frightening sounds, such as the breaking of a tree branch or some animal passing by. In such a situation, his practice took the following form:

Brahmin, that fear and dread came upon me while I was walking. Then, brahmin, I did not stand, nor sit, nor lie down, until I had dispelled that fear and dread while walking. Brahmin, that fear and dread came upon me while I was standing. Then, brahmin, I did not walk, nor sit, nor lie down, until I had dispelled that fear and dread while standing. Brahmin, that fear and dread came upon me while I was sitting. Then, brahmin, I did not lie down, nor stand, nor walk, until I had dispelled that fear and dread while sitting. Brahmin, that fear and dread came upon me while I was lying down. Then, brahmin, I did not sit, nor stand up, nor walk, until I had dispelled that fear and dread while lying down.

(MN 4: *tassa mayhaṃ, brāhmaṇa, caṅkamantassa taṃ bhayabheravaṃ āgacchati. so kho ahaṃ, brāhmaṇa, n’ eva tāva tiṭṭhāmi na nisīdāmi na nipajjāmi, yāva caṅkamanto va taṃ bhayabheravaṃ paṭivinemi. tassa mayhaṃ, brāhmaṇa, ṭhitassa taṃ bhayabheravaṃ āgacchati. so kho ahaṃ, brāhmaṇa, n’ eva tāva caṅkamāmi na nisīdāmi na nipajjāmi, yāva ṭhito va taṃ bhayabheravaṃ paṭivinemi. tassa mayhaṃ, brāhmaṇa, nisinnassa taṃ bhayabheravaṃ āgacchati. so kho ahaṃ, brāhmaṇa, n’ eva tāva nipajjāmi na tiṭṭhāmi na caṅkamāmi, yāva nisinno va taṃ bhayabheravaṃ paṭivinemi. tassa mayhaṃ, brāhmaṇa, nipannassa taṃ bhayabheravaṃ āgacchati. so kho ahaṃ, brāhmaṇa, n’ eva tāva nisīdāmi na tiṭṭhāmi na caṅkamāmi, yāva nipanno va taṃ bhayabheravaṃ paṭivinemi*.).

If fear and dread came [while] I was walking, then at that time I did not sit down or else lie down, determining to discard the fear and dread, and [only] afterwards did I sit down. If fear and dread came while I was standing, then at that time I did not walk and also did not sit down, determining to get that fear and dread discarded, and [only] afterwards did I sit down. If fear and dread came while I was sitting, I did not [stand up or] walk, determining to discard the fear and dread, and [only] afterwards did I walk. If fear and dread came while I was lying down, then at that time I did not sit up and also did not walk, determining to get that fear and dread discarded, and [only] afterwards did I sit up.

(EĀ 31.1: 若我經行有畏怖來者, 爾時我亦不坐臥, 要除畏怖, 然後乃坐. 設我住時有畏怖來者, 爾時我亦非經行, 亦復不坐, 要使除其畏怖, 然後乃坐. 設我坐時有畏怖來者, 我不經行, 要除畏怖, 然後乃行. 若我臥時有畏怖來者, 爾時我亦非經行, 亦復不坐, 要使除其畏怖, 然後乃坐; the original text has confused some of the bodily activities, which have been corrected based on variant readings).

This description, given in similar ways in the two parallel versions, points to mindfulness of bodily postures, even though mindfulness is not explicitly mentioned. The potential to face fear in this way would be related to the type of grounding in the here and now that such deployment of mindfulness can provide. Being seated alone in a forest and hearing an unexpected sound, the tendency to react immediately is in a way natural. Yet, acting out that impulse is a way of succumbing to the fear and reacting to it, rather than facing anxiety calmly for what it is. Of course, fear can at times have an important function. But the type of noise described in the discourse does not give the impression that there was an urgent need for the Buddha-to-be to protect himself. Instead, the episode appears to be just about hearing the kind of noise that naturally occurs in a forest. Some branch breaks off somewhere; somewhere else an animal passes by and one hears its steps, etc.

Understood in this way, the passage can be taken to exemplify quite vividly a type of attitude that is indeed salient in mindfulness training, namely, the ability to remain aware of what happens without immediately reacting to it. In this way, the mind’s tendency to build up apprehensions of what will happen next is countered, and one learns instead to stay with what is happening right now. This offers a middle way approach to fear and anxiety that avoids the two extremes of suppression and surrender. As pointed out by Giustarini (2012, p. 529) in a comment on the above description, “physical stillness and stability seem to be able to inspire mental stillness and stability, which are helpful remedies in counteracting and eventually eradicating fear.” In fact, according to early Buddhist thought, fully awakened ones are completely free from fear; at the same time, they are constantly in the possession of mindfulness (AN 8.28, SĀ 694, and Up 6067; Anālayo 2020).

## Fear and COVID-19

The potential to learn to face fear without either repressing it or else acting it out is particularly relevant in view of the current COVID-19 virus disease, whose repercussions in a range of different sections of society have led to a proliferation of fear and anxiety. The magnitude and quick spread of the pandemic threatens to result in mental destabilization of a considerable part of the population worldwide, resulting in feelings of helplessness in view of the sometimes drastic changes in living situations, the breakdown of social structures long taken for granted, an overall sense of isolation, and of course the ultimate fear: death. These effects, which tend to be further increased by social contagion when anxiety spreads from one person to another, can range from depression and panic attacks to post-traumatic stress disorder symptoms, psychosis, and even suicidality.

In view of this situation, mindfulness practice can offer a tool to help face fear and anxiety (Goyal et al. 2014; Gu et al. 2015; Hoge et al. 2013; Roy et al. 2020; Spijkerman et al. 2016). Judging from the extract translated above, the commendable form of mindfulness practice could in particular be the cultivation of proprioceptive awareness of one’s bodily postures. The main task, when experiencing the arising of fear, would be to return to being mindful of one’s present bodily posture and try to relax into the resultant embodied presence of the mind. Mindfulness of the breath as part of awareness of the whole body could become part of this grounding in the experience of the body, as long as such directing of attention to the breath is not undertaken as an exclusive focus but rather as an integral part of awareness of the whole body.

An important dimension of mindfulness of the body, in the way this is described in the early discourses, is its ability to lead to a broad state of mind. The early Buddhist discourses employ the term “boundless” to qualify the mind of one who has established mindfulness of the body, thereby using a same term that usually designates the immeasurables (*appamāṇa*, *apramāṇa*, 無量, *tshad med pa*) or *brahmavihāra*s (Anālayo 2019a). From a practical perspective, this implies that the cultivation of mindfulness of one’s postures should be undertaken in such a way that one’s mind becomes broad and open rather than narrow and closed. The difference that emerges in this way is particularly pertinent to the experience of fear, which can easily lead to a narrow state of mind and a closing down rather than an opening up.

The stress resulting from fear tends to diminish resilience (McEwen 2016), which is of course particularly needed during the spread of a highly infectious disease like COVID-19. Here, mindfulness practice might offer a tool to strengthen the immune system (Kaliman et al. 2014; see also Conklin et al. 2018 on the effect of intense mindfulness meditation on telomere biology). From this perspective, facing fear with mindfulness can be expected to have not only immediate (and long-term) beneficial effects on the mind but also on the body, in line with a recurrent pattern evident in some of the material surveyed above.

## Abbreviations

AN: *Aṅguttara-nikāya*
EĀ: *Ekottarika-āgama* (T 125)
MĀ: *Madhyama-āgama* (T 26)
MN: *Majjhima-nikāya*
SĀ: *Saṃyukta-āgama* (T 99)
SĀ^2^: *Saṃyukta-āgama* (T 100)
SN: *Saṃyutta-nikāya*
Sn: *Sutta-nipāta*
T: Taishō edition
Up: *Abhidharmakośopāyikā-ṭīkā*
Vin: *Vinaya*
Vism: *Visuddhimagga*
